# The impact of antihypertensives on kidney disease

**DOI:** 10.12688/f1000research.9916.1

**Published:** 2017-05-02

**Authors:** Diego F Marquez, Gema Ruiz-Hurtado, Luis Ruilope

**Affiliations:** 1 Unidad de Hipertensión Arterial, Servicio de Medicina Clínica, Hospital San Bernardo, Salta, Argentina; 2 Instituto de Investigación Imas12 and Unidad de Hipertensión, Hospital 12 de Octubre, Madrid, Spain; 3 Departamento de Medicina Preventiva y Salud Pública, Universidad Autónoma, Madrid, Spain; 4 Escuela de Estudios Postdoctorales and Investigación, Universidad de Europa de Madrid, Madrid, Spain

**Keywords:** arterial hypertension, chronic kidney disease, CKD, antihypertensives, kidney disease

## Abstract

Arterial hypertension and chronic kidney disease (CKD) are intimately related. The control of blood pressure (BP) levels is strongly recommended in patients with CKD in order to protect the kidney against the accompanying elevation in global cardiovascular (CV) risk. Actually, the goal BP in patients with CKD involves attaining values <140/90 mmHg except if albuminuria is present. In this case, it is often recommended to attain values <130/80 mmHg, although some guidelines still recommend <140/90 mmHg. Strict BP control to values of systolic BP around 120 mmHg was recently shown to be safe in CKD according to data from the SPRINT trial, albeit more data confirming this benefit are required. Usually, combination therapy initiated with an angiotensin receptor blocker (ARB) or angiotensin-converting enzyme inhibitor (ACEi) and commonly followed by the addition of a calcium channel blocker and a diuretic is needed. Further studies are required as well as new drugs in particular after the positive data obtained from new oral anti-diabetic drugs.

## Introduction

Clinical and epidemiological evidence has demonstrated the importance of blood pressure (BP) control in order to prevent the development and to reduce the progression of chronic kidney disease (CKD) ^1^. On the other hand, CKD is accompanied by a very significant increase in cardiovascular (CV) risk ^2^. As a consequence, the treatment of arterial hypertension is simultaneously directed to promote renal and CV protection. It is widely accepted that suppression of the renin-angiotensin-aldosterone system (RAAS) through the use of an angiotensin-converting enzyme inhibitor (ACEi) or an angiotensin receptor blocker (ARB) constitutes the first step of therapy in patients with CKD ^1^.

Studies examining the effect that antihypertensives have on kidney function have typically involved early or pre-existing primary renal disease and/or diabetic nephropathy patients. These trials were devoted primarily to investigating the evolution of renal function during a short period of time and in relatively small samples that impeded our knowledge of the simultaneous effects on CV outcome ^3^. Initially, the capacity of ACEi and ARB for the diminution and primary prevention of albuminuria in CKD, and particularly in type 2 diabetics, was investigated ^3– 5^. The changes in albuminuria together with the control of arterial hypertension were determinants for the improvement in renal outcomes ^3^. However, recent data have also shown that in hypertensive CKD patients with low levels of albuminuria, the exclusive attainment of low BP goals is associated with an improvement in eGFR ^6^. Thus, in CKD patients, the presence of albuminuria is considered in KDIGO and several other guidelines as the key point to recommend a stricter BP goal (≤130 mmHg systolic and ≤80 mmHg diastolic if albumin excretion rate is ≥30 mg/24 hours) ^7, 8^. A similar BP goal may be considered in patients with low or no level of albuminuria. In fact, the renal data of the SPRINT trial indicate that a SBP goal lower than 120 mmHg was safe and effective in a subgroup of patients with CKD ^9^.

## Hypertension as a mediator of the correlation between chronic kidney disease and cardiovascular disease

Albuminuria, increased serum creatinine values, and a lower eGFR are considered by current arterial hypertension guidelines as significant CV risk factors that add to the already-high risk of pre-existing CV risk factors such as diabetes, cholesterol, and smoking ^10, 11^. In fact, patients developing end-stage renal disease (ESRD) and entering dialysis are a minority among the CKD population; one could also consider them to be survivors, as a large number of patients with CKD die from CV disease before ESRD develops. As can be seen in the article published by Levey *et al*. ^12^ with data from the USA, 4.9% of the total CKD population has an eGFR below 60 mL/minute/1.73 m ^2^. This represents a very relevant number of patients with CKD in stages 3a, 3b, 4, and 5, but only 0.2% attain the stage ^12^. These data are obtained from different surveys (NHANES) performed in the US, and since these are cross-sectional data it does not capture the fact that many patients die before reaching the terminal stages of CKD. Recently, we published a review ^13^ showing that data from some clinical trials in the field of arterial hypertension have demonstrated that CV and renal protection can be attained simultaneously using the same therapy, mainly consisting of a renin-angiotensin-aldosterone system (RAAS) blocker, a calcium antagonist, and a diuretic, to which a statin is frequently added. These data indicate that early and aggressive control of hypertension and albuminuria is very important to simultaneously protect the CV and renal systems.

Unfortunately, many patients attain the advanced stages of CKD, where RAAS blockade is often incomplete in a relevant percentage of patients, particularly in stages 3b and 4 of CKD, not tolerating high doses owing to the frequent development of hyperkalemia. The recent development of new potassium binders will contribute to facilitating the long-term maintenance of RAAS blockers, in particular mineralocorticoid receptor antagonists, while maintaining adequate serum potassium levels ^14, 15^.

## Antihypertensive therapy, blood pressure goals, and renal function

Different mechanisms play a key role in the development and maintenance of hypertension in CKD. The most important of them are volume overload, due to the difficulty in sodium and water handling, and activation of the RAAS and of the sympathetic nervous system ^2^. These effects influence intraglomerular pressure negatively through the facilitation of the pulsatile transmission of uncontrolled systemic BP ^16^. The renoprotection provided by antihypertensive agents depends on their capacity to lower systemic BP, impeding the damage in the renal vasculature from the arteries to the renal microcirculation and to the glomeruli through their specific effects on renal hemodynamics ^16^. Usually, the combination of more than two drugs is needed to attain an adequate control that depends on counteracting the three main factors intervening in the development and progression of hypertension.

The goal BP for CKD is commonly recommended to be below 140/90 mmHg, except when albuminuria is presented. The abandoning of the prior commonly recommended BP goal of <130/80 mmHg in patients with CKD was due to the failure of randomized controlled trials (RCTs) devoted to investigating strict BP control in CKD to show benefit, particularly the Modification of Diet in Renal Disease (MDRD) study ^17^, the BP Control for Renoprotection in Patients with Nondiabetic Chronic Renal Disease (REIN-2) trial ^18^, and the African American Study of Kidney Disease (AASK) ^19^. A secondary analysis of the AASK trial reported that strict BP control in patients with albuminuria >0.22 g/g creatinine had fewer composite endpoints (HR 0.73, p=0.01) ^20^, confirming the adequacy of lower goal BP with albuminuria. Interestingly, a long-term combined analysis of the MDRD and AASK studies showed that strict BP control did not delay the onset of ESRD but did reduce the relative risk of death in patients with CKD (0.87, 95% confidence interval [CI] 0.76–0.99) during long-term follow up ^21^.

On the other hand, data published as a meta-analysis of 11 randomized controlled trials in non-diabetic renal disease patients demonstrated that an SBP of 110–129 mmHg was associated with a lower risk for progressive renal disease ^22^. Interestingly, increased renal risk was observed with SBP values of <110 mmHg; this is in line with the potential negative effects on the kidney caused by a significant drop in renal perfusion pressure after the consequences of a maintained elevation of BP and the development of nephrosclerosis have taken their toll on the renal vasculature ^23^. Other data related to mortality in patients with CKD indicate that the optimal BP differs and oscillates between 130 and 159/70 and 89 mmHg ^24^. The progression of diabetic and non-diabetic renal disease was more effectively slowed down, and survival was also improved, when patients were administered an antihypertensive regime that included an ACEi or an ARB compared to a regime without; this observation was independent of attained BP ^25^. On the other hand, dual blockade of the RAAS using ACEi plus ARB simultaneously did not show benefits in large-scale trials ^26– 28^. In the same way, the addition of the direct renin inhibitor aliskiren to standard therapy with a RAAS blocker in high CV risk patients with diabetes is not supported and may even be harmful ^28^. As a consequence, combined use of the two RAAS blockers in patients with CKD has been discarded by guidelines ^8, 29^.

Data from the Action to Control Cardiovascular Risk in Diabetes (ACCORD) study ^30^ indicate that strict BP control (SBP <120 mmHg) did not improve renal outcomes of type 2 diabetic patients, and according to several reports the SBP goal of <130 mmHg remains adequate in this condition for renal and CV protection ^13, 31^.

## Blockade of aldosterone: an unfulfilled promise?

Aldosterone is a relevant mediator of progressive renal and CV damage ^32^. It is well known that aldosterone antagonists reduce albuminuria in patients with CKD, on top of a RAAS blocker, by an amount of 30–40% ^33– 35^. As previously mentioned, a limiting factor for the wide use of aldosterone antagonists is linked with the risk of inducing hyperkalemia, which becomes a prominent concern as renal function deteriorates. Finerenone is a novel nonsteroidal mineralocorticoid receptor antagonist (MRA) that has greater receptor selectivity than spironolactone and eplerenone, can significantly decrease albuminuria in diabetic nephropathy, and has a much lower prevalence of hyperkalemia ^36^. Actually, two seminal studies are ongoing to test the capacity of the new MRA finerenone to slow down the progression of diabetic nephropathy (NCT02540993) ^37^ and to protect the CV system in type 2 diabetic patients (NCT02545049) ^38^. The use of the new potassium binder patiromer and the selective cation exchanger sodium zirconium cyclosilicate ^15^ will contribute to the facilitation of MRA use in patients with CKD.

## Evidence with other therapies and renal outcomes

Recently, new data on the role of sodium intake in the progression of CV disease ^39^ and renal disease ^40– 42^ have been published, enhancing the need for an adequate intake of sodium in patients with CKD. A low-sodium diet contributes to the reduction of albuminuria by itself and through the enhancement of RAAS blockade ^40, 41^. On the other hand, there are drugs other than hypertensives that can positively influence renal function together with BP control, such as statins ^43^, fibrates ^44^, thiazolidinediones ^45^, and antiplatelet therapy ^46^. Treatments such as allopurinol ^47^ and vitamin D supplementation ^48^ require further investigation.

Recently, the results of the EMPA-REG trial showed a lower rate of CV death, total death, and heart failure in type 2 diabetic patients with the addition of empagliflozin to standard care ^49^. Moreover, the analysis of secondary end points of the EMPA-REG trial showed that empagliflozin was associated with a slower progression of CKD ^50^. These good results were partially due to the antihypertensive and natriuretic capacities of the drugs ^51^.

Results obtained with GLP-1 agonists liraglutide ^52^ and semaglutide ^53^ have also shown a significant improvement in CV outcomes of type 2 diabetics that was accompanied by a more significant decrease in body weight and a less important drop in BP in the absence of natriuretic effects. This last point could explain the absence of effects on heart failure. Liraglutide also exhibited a renal protective capacity through a significant decrease in albuminuria ^52^. Further studies will be presented in the near future with these two classes of drugs and will contribute to the reconsideration of type 2 diabetes treatment with regard to CV and renal protection.

In summary, strict BP control, adequate suppression of the RAAS, and an integrated approach to protection of the increased global CV risk are required to prevent the appearance and progression of CKD and simultaneously of CV disease. New drugs are coming that could facilitate the control of BP and improve both renal and CV outcomes.
